# Supplementary material for: Are carbon emissions trading and green financial instruments synergistic? -Comprehensive quantitative research based on content analysis
Source: PLoS One. 2024 Mar 7;19(3):e0298601. doi: 10.1371/journal.pone.0298601 (PMC10919601; doi:10.1371/journal.pone.0298601)
Supplement: S1 Data — (ZIP) [file pone.0298601.s001.zip › Data/地级市Z_F工作报告.zip!/╕╩╦α/╠∞╦«╩╨/└┤╫╘╣1⁄2╓┌║┼í╛┬φ┐╦╩2╛▌═°í┐_2018.docx]

各位代表:

现在,我代表市人民政府向大会报告工作,请予审议,并请各位政协委员和其他列席人员提出意见。

2017年政府工作回顾

2017年,在省委、省政府和市委的坚强领导下,市政府深入贯彻党的十八大和十八届三中、四中、五中、六中全会精神,认真学习贯彻党的十九大精神和习近平新时代中国特色社会主义思想,全面落实习近平总书记视察甘肃重要讲话和“八个着力”重要指示精神,按照省第十三次党代会和市第七次党代会部署要求,团结带领全市干部群众,负重奋进、争先进位、主动作为、扎实工作,全市经济社会持续健康发展,较好地完成了市七届人大一次会议确定的各项目标任务。预计实现生产总值644亿元,同比增长5.5%,其中,第一产业增加值110.5亿元,增长5.8%;第二产业增加值207.5亿元,增长5%;第三产业增加值326亿元,增长6%。规模以上工业增加值112亿元,增长7%。固定资产投资675亿元,增长0.6%。社会消费品零售总额316亿元,增长9.5%。一般公共预算收入44.7亿元,同口径增长10.95%。城镇居民人均可支配收入24725元,增长9%;农村居民人均可支配收入7149元,增长10%。安全生产、节能减排和环境质量指标均在控制目标以内。

重大项目建设进展良好。争取国家和省上预算内投资项目124个,落实资金8.9亿元。实施招商引资项目351个,到位资金408亿元,27个重点招商引资项目完成投资57.3亿元;第23届兰洽会签约项目97个,拟引进投资320.5亿元。81个省管市列重大项目和200个市级领导包抓项目分别完成投资150.23亿元和285.24亿元。实施农业重点项目17个,完成投资18.39亿元;实施工业重点项目19个,完成投资14.52亿元;实施城建重点项目153个,完成投资116亿元;实施社会事业重点项目16个,完成投资20.64亿元。向社会重点推介PPP项目45个,总投资1107.8亿元。宝兰客专、麦甘公路建成通车,天水南站综合交通枢纽及站前广场中轴线和中区旅客通道、宝兰客专秦安站枢纽工程、天水火车站站房改造、天水汽车西站、甘谷客运中心基本建成,陇海铁路元龙段改线工程顺利实施,天平高速公路天水段启动建设,甘肃(天水)国际陆港发展战略规划获省政府批复,天水军民合用机场迁建项目省政府与西部战区已正式签订协议、预可研报告即将批复。引洮二期工程天水段、城区引洮供水、曲溪城乡供水工程净水厂及输配水管网、秦州区关峡水库、秦安县小湾河水库、张家川县富川水库、清水县城区供水等项目加快实施。酒泉至湖南±800千伏特高压直流输电工程天水段、宝兰客专330千伏供电工程全面建成。

精准扶贫脱贫攻坚成效显著。建立深度贫困对象市、县区、乡镇三级联系帮扶责任制,深入实施精准扶贫精准脱贫方案和“七个一批”工程,全年预计减贫12万人,126个贫困村实现整村脱贫。争取国家和省上专项扶贫资金9.83亿元、东西扶贫协作帮扶资金6514.8万元,市、县区财政投入专项扶贫资金8560万元,落实扶贫互助资金及“双助贷”资金20亿元,贫困县统筹整合财政涉农资金16.55亿元。新建改建农村公路1215.5公里,硬化村组道路341.93公里,硬化巷道54.31万平方米。改造农村D级危房15011户,建成易地扶贫搬迁安置点86个,搬迁贫困人口4163户20021人。新建贫困村幼儿园89所、标准化村卫生室205个,改造提升“乡村舞台”705个,贫困村4G互联网、快递服务网点、电子商务站(点)实现全覆盖。为农村困难群众发放低保和救助资金7.95亿元,全市“三户一孤”人员实现大病救助保险全覆盖。

特色产业发展步伐加快。实施现代农业推进计划和“一十百千”现代农业建设工程,粮食总产113.28万吨,增长0.98%。新增果品基地15.4万亩、蔬菜面积2.26万亩、标准化规模养殖场(小区)82个,果品、蔬菜、畜牧业产值分别达到103亿元、80亿元、77.4亿元,输转农村劳动力69.25万人,创劳务收入128.5亿元。10个省、市级现代农业示范园区累计完成投资51亿元,100个科学发展示范点全面建成,新培育农业产业化龙头企业26家,新注册农民合作社1051家、家庭农场234家。新流转农村土地8.2万亩,流转总面积达到113.6万亩,流转率20.6%。深入推进工业“扩量提质”工程,华天电子科技园、长城电工产业园基本建成,岐黄中医药产业园主体完工,170户规模以上企业实现利润12.5亿元,增长25%,27户战略性新兴产业企业增加值增长20%。军民融合产业园规划上报待批,通用航空产业规划编制有序推进,装配式建筑产业园启动建设,与国药集团、上海医药集团、浪潮集团签订战略合作协议。天水经济技术开发区冀城产业园启动建设,7户出城入园企业完成搬迁改造。宏基环保、翔达颜料在“新三板”挂牌。麦积山大景区、大地湾国家考古遗址公园、甘谷毛家坪遗址开发保护有序推进,麦积区、武山县国家全域旅游示范区创建工作成效明显,秦州区被命名为全国休闲农业和乡村旅游示范县,天水66号文化创意园被文化部确定为“双创”示范基地。预计旅游接待3711万人次、旅游综合收入212亿元,均增长33.5%;文化产业实现增加值17.15亿元,增长20%。天水会展中心建成试运营,天水商贸城、甘谷物流中心、秦安小商品城、武山洛门蔬菜批发市场二期、天水市菜篮子工程麦积(仓储)配送中心、秦州区果品市场高端库项目建设进展顺利。秦安县、甘谷县被确定为“国家电子商务进农村综合示范县”,预计电子商务交易额35亿元,增长20%。

城乡环境面貌变化明显。加强城乡基础设施建设,藉河生态环境综合治理一期续建、秦州热源厂及亚行贷款城区供热管网一期、省建投天水总部经济城一期、天润桥、天秀桥、秦安葫芦河2号大桥全面建成,成纪新城地下净水厂、固体垃圾处理循环产业园、二十里铺大桥及连接线、西关片区古城综合保护等项目积极推进,城市建成区面积达到68平方公里。完成豹子沟等8条黑臭水体治理,改造城区小巷道105条、人行道18.14万平方米,改造提升公交站点280个,新建停车场14处,新增停车位7252个。麦积区被确定为全国新型城镇化综合试点县区。完成营造林26.5万亩,建设堤防67.3公里,新修梯田12万亩,新增高效节水灌溉面积4万亩,治理水土流失面积252平方公里,改造提升农村饮水安全工程26处、惠及24.53万农村人口,实施土地整治项目21个15.8万亩,完成地质灾害治理项目13个,建成“美丽乡村”省级示范村20个、市级示范村25个、市级环境整洁村305个。电信普遍服务试点项目全面完成,全市行政村光纤网络实现全覆盖。加强城市管理创新,智慧城市运营中心建成使用,“全国文明城市”“国家园林城市”创建工作成效明显。全面加强环境保护工作,扎实开展中央环保督察反馈问题整改,建立健全“河长制”责任体系,开展全域无垃圾治理活动,武山、秦安、清水、张家川城区生活垃圾填埋场通过无害化等级评定,全市城区生活垃圾无害化处理率达100%。

人民生活水平稳步提高。省列10项、市列12件民生实事全面完成。市一中麦积校区、麦积天润学校建成招生,市三中迁建项目有序推进,市二中、启升中学、甘谷一中、秦安五中创建为全省第三批“普通高中特色实验学校”。实施“全面改薄”项目579个、完成投资8.01亿元,秦州、麦积、甘谷通过国家义务教育基本均衡县评估验收,秦安陇城教育园区办学模式得到中央媒体好评。普通高考上线人数增长4.27%,上线率提高1.16个百分点。争取国列、省列科技计划项目45项,组织实施科技项目417项,取得科技成果109项,新申报省级工程技术中心2个,新认定和重新认定高新技术企业19家。市体育中心加快建设,建成街道综合文化站7个、社区综合文化中心75个、乡镇及社区体育健身中心8个、行政村体育惠民工程180个,我市全景拍摄的电视剧《苦乐村官》在央视热播。城市公立医院综合改革全面启动,实现了市级城乡居民医疗医保整合和就医异地结算,市中医院入选国家中医药传承创新工程重点建设单位。落实计划生育“二孩”政策,人口自然增长率6.14‰。新增城镇就业5.46万人,城镇登记失业率3.12%。全面实施全民参保登记计划,“五险合一”上线运行,城乡居民社会养老保险参保率96.91%。城乡低保对象实现动态管理下的应保尽保,城市低保标准提高8%,农村低保一、二类对象补助水平分别提高到3500元和3300元。建成城市社区老年人日间照料中心26个、农村互助老人幸福院130个。新开工棚户区改造2.19万户,建成棚户区改造安置住房和公租房4800套,发放住房租赁补贴13850户2053.9万元。加强社会治安综合治理,及时调处化解矛盾纠纷和信访问题,强化食品药品安全监管和重点行业领域安全隐患排查整治,加强民族宗教工作,社会大局保持和谐稳定。统计调查、外事侨务、扶残助残、防震减灾、档案史志、法律服务、气象、人防、仲裁、住房公积金等方面工作在服务经济社会发展中发挥了积极作用。妇女儿童、老年人和关心下一代等工作取得新成绩。

改革开放扎实有效推进。加快“接放管服”改革,当年取消调整和下放行政审批事项9项。全面实施“多证合一”和企业简易注销制度,新增市场主体10177户。机关单位公车改革全面完成,市级公务用车平台建成运行。深化国资国企改革,明确市属国有企业功能定位,完成5户企业“三供一业”分离移交,盐业体制改革积极推进。农村土地承包经营权确权登记颁证全面完成,国有林场改革和经济林确权登记颁证有序开展,土地“三权”分置、农村集体产权制度改革和第三次全国农业普查进展顺利,农村“三变”改革扎实推进,国有建设用地使用权二级市场改革试点和不动产统一登记工作稳步推进。甘谷县供销社综合改革通过国家验收,“三社合一”“双线运行”试点经验在全省推广。哈尔滨银行、长安银行在我市设立村镇银行,中国银联甘肃分公司陇东业务部落户天水,金融质量服务年活动成效明显。成功举办公祭伏羲大典暨第28届伏羲文化旅游节、2017绿色及可持续发展麦积山论坛和装配式建筑产业论坛、大健康产业论坛、西北蔬菜产业绿色发展武山论坛、中国·天水李杜诗歌节、花牛苹果宣传推介活动,承办了中国山地自行车公开赛、中国龙舟公开赛、中国垂钓电视直播精英赛、第四届国际城市围棋公开赛、第三届“李广杯”国际射箭锦标赛、第16届环湖赛天水至平凉赛段等体育赛事,麦积区被评为全国群众体育先进单位。开通天水至西安、重庆、天津、南京、杭州等航线。兰州海关驻天水办事处挂牌运行,预计完成进出口总额32亿元。

政府自身建设不断加强。坚持依法治市,“七五”普法深入推进。积极推进地方立法工作,《天水市烟花爆竹安全管理办法》发布实施,《天水市饮用水水源保护区环境保护管理办法》正在修改完善。自觉接受市人大及其常委会法律监督和工作监督、市政协民主监督、社会各界广泛监督,225件人大代表意见建议、242件政协提案全部办结。建立落实市政府党组会、常务会会前学习制度,推动“两学一做”学习教育常态化制度化。修订完善了市政府党组会议、中心组学习会议、常务会议、市长办公会议、市长碰头会议等制度。全面加强政府系统党的建设,定期向市委常委会汇报工作,认真落实党风廉政建设主体责任和领导干部“一岗双责”,加强行政监察、审计监督和政府督查,扎实开展“三纠三促”“两查两保”专项行动和“明查暗访督查年”活动,行政效能和政府执行力不断提升。

各位代表,在充分肯定成绩的同时,我们也清醒地认识到,市政府工作还存在一些问题和不足。主要是:虽然主要经济指标增速位居全省前列,但生产总值、固定资产投资等经济指标没有达到市人代会确定的预期目标,有些指标欠账较大;项目建设还不能适应新形势新任务的要求,谋划储备的大项目特别是产业化项目较少,项目前期准备不够充分,个别项目没有如期开工,有的在建项目进度缓慢,项目审批环节多、周期长,投资发展软环境还不够好,招商引资项目落地见效不够理想;脱贫攻坚任务艰巨,尤其是深度贫困地区基础条件差,内生动力不足,一些边远地区群众产业、吃水、行路、住房等问题还没有得到很好解决,帮扶责任落实不到位,有的政策措施还没有见到实效;城市基础设施建设相对滞后,停车难、交通拥堵、一些地方乱搭乱建问题较为突出,城市管理科学化精细化水平有待提高;县域经济发展相对缓慢,经济总量偏小、产业支撑不强、特色不够明显、发展活力不足;社会事业发展不平衡不充分,城区中小学“大班额”现象比较突出,卫生、文化、科技、体育等公共服务设施欠账较多,基本服务还不能完全满足群众日益增长的美好生活需要;大气、水、土壤污染防治压力较大,安全生产、社会治安和食品药品监管等领域还存在一些薄弱环节;一些政府工作人员服务意识较差,思想不够解放,作风不实、担当不够、效率不高,不作为、慢作为问题比较突出,乱作为现象时有发生,持续改进作风、优化发展环境任务仍然艰巨。对于这些问题和不足,我们将高度重视,努力加以解决。

各位代表,过去一年的成绩来之不易,这是省委、省政府正确领导、大力支持的结果,是市委统揽全局、坚强领导的结果,是市人大及其常委会、市政协和社会各界倾力支持、有效监督的结果,是全市干部群众齐心协力、团结奋斗的结果。在此,我代表市人民政府,向各位人大代表、各位政协委员,向奋战在全市各条战线上的广大干部群众,向各民主党派、人民团体、工商联和无党派人士,向离退休老同志,向天水籍在外人士,向驻市人民解放军、武警消防官兵和中央、省属驻市单位,表示崇高的敬意和衷心的感谢!

2018年政府工作的主要任务

2018年,是全面贯彻党的十九大精神的开局之年,也是加快实施“十三五”规划和打赢脱贫攻坚战、决胜全面建成小康社会的关键一年。政府工作的总体思路是:认真学习贯彻党的十九大和省第十三次党代会精神,以习近平新时代中国特色社会主义思想为指导,全面落实习近平总书记视察甘肃重要讲话和“八个着力”重要指示精神,抢抓国家推进“一带一路”等重大机遇,牢固树立和贯彻新发展理念,坚持稳中求进、争先进位的工作总基调,紧紧围绕高质量发展的根本要求,坚定不移实施工业强市战略,以推进供给侧结构性改革为主线,以脱贫攻坚为统揽,以项目建设为抓手,以作风建设为保障,统筹抓好稳增长、促改革、调结构、惠民生、防风险各项工作,努力推动经济社会持续健康发展,更好地满足全市人民日益增长的美好生活需要。

全市经济社会发展的主要预期目标是:生产总值增长6.5%,规模以上工业增加值增长7%,固定资产投资增长6%,社会消费品零售总额增长9%,一般公共预算收入增长7%,城镇居民人均可支配收入增长9%,农村居民人均可支配收入增长10%,居民消费价格指数涨幅控制在3%以内,环境质量和节能减排指标均控制在省上下达目标以内。

围绕上述目标,市政府将重点抓好以下八个方面的工作:

一、强化项目支撑,着力夯实经济社会发展基础

牢固树立抓项目就是抓发展、抓发展必须抓项目的理念,认真落实“三重”“三一”要求,坚持续建项目抓进度、新建项目抓开工、储备项目抓前期、招商项目抓落地、所有项目抓投资,充分发挥项目在扩投资、稳增长、调结构、强基础、促发展、惠民生中的重要作用。

立足产业谋划项目。在继续提升优化原有传统产业和优势产业的基础上,立足实际,超前谋划,积极探索培育新型战略性产业。抢抓宝兰高铁开通重大机遇,立足我市资源优势和基础条件,把文化旅游作为全市首位产业,紧紧围绕文化、旅游、体育、康体、养生、养老等,着力发展大文化、大旅游、大健康产业,规划建设好伏羲文化旅游产业园、麦积山文化旅游产业园、大地湾文化旅游产业园、卦台山文化旅游产业园等,力争旅游接待突破4000万人次,旅游综合收入达到240亿元。抓住国家和省上大力发展通用航空业的政策机遇,谋划发展以飞机维修和培训、无人机生产、航空食品等为重点的航空产业。紧抓我市被确定为全省装配式建筑试点城市机遇,大力发展以钢结构、混凝土预制、木器加工、建筑配件等为主的装配式建筑产业。依托国药集团、上海医药集团、重庆太极集团、深圳翰宇药业等,充分发挥我市岐黄药业、成纪药业、天森药业等企业资源优势,建立以连翘、半夏等为主的特色中药材种植基地,大力发展中成制药和生物制药,做大做强医药保健产业。加强与浪潮集团等企业的合作,加快建设集互联网、物联网、云计算、大数据为一体的智慧城市和信息产业园,大力发展信息产业。

加大力度实施项目。开工建设麦积山游客服务中心、华昌欢乐城、天水大剧院、长城果汁年产10万吨果蔬汁生产线、6913北斗卫星通讯模块生产、海林中科产业升级、华建新材料公司年处理100万吨建筑垃圾等项目。启动实施甘肃(天水)国际陆港基础设施建设、天水机场迁建试验段建设。建成天定高速藉口出口、G310线秦州至武山段、秦州大道、麦积山大道、甘泉至麦积山景区旅游公路、洛门至水帘洞旅游公路、潘集寨至温泉旅游公路,开工建设S25静天高速公路、陇县至榜罗公路秦安至甘谷礼辛段、天水南站综合交通枢纽及站前广场二期、天水南站新增动车组存车配套设施等项目,全力保障天平高速公路天水段建设,积极推进天水绕城高速公路、G310线秦州麦积城区段改线提升工程前期工作。加快重点水利项目建设,继续抓好引洮二期天水配套、城区引洮供水等工程,开工建设曲溪城乡供水项目。

优化环境引进项目。在积极向上汇报争取项目的同时,加大招商引资力度,依托天水经济技术开发区和各级各类园区平台,积极引进“补链”“强链”“延链”项目,促进产业转型升级。建立完善引进项目、资金、人才等的优惠政策,落实重点招商项目落地定期通报制度,对已签约项目紧盯不放、跟踪衔接,推动项目尽快落地实施。全面开展“接放管服”突破年活动,切实解决项目审批环节多、资料多、时间长等问题。扎实开展营商环境建设年活动,强化“你投资我服务、你发财我发展”的理念,积极主动热情为客商服务,构建“亲”“清”新型政商关系,进一步营造重商亲商安商富商的良好环境。

强化保障推进项目。完善项目包抓机制,坚持市级领导包抓重大项目制度,做到一个项目、一名领导、一套班子、一个方案、一抓到底,实行“一季度一观摩一评比”,推动项目加快实施。完善投资融资机制,引导金融机构加大信贷支持,积极推广运用PPP模式,紧盯重点推介洽谈的45个项目,主动对接有意向的社会资本,保障项目建设资金需求。完善并联审批机制,定期开展重大项目联审联批,推行投资项目在线审批。完善监督管理机制,抓好工程施工质量、安全生产监管、项目资金审计、廉政风险防控等工作,努力建设优质、安全、放心、满意工程。

二、聚焦精准脱贫攻坚,着力提升“三农”工作水平

积极实施乡村振兴战略,按照“产业兴旺、生态宜居、乡风文明、治理有效、生活富裕”的总要求,坚持以脱贫攻坚统揽农业农村工作全局,强化“一号工程”意识,切实下足“绣花”功夫,精细精确精微抓重点、补短板、强弱项,确保全年减贫10万人以上,武山县、甘谷县脱贫摘帽。

紧盯深度贫困区域抓攻坚。把深度贫困地区脱贫攻坚摆在突出位置,集中各方面力量,采取超常规措施,啃硬骨头,打攻坚战,下功夫解决好贫中之贫、坚中之坚、难中之难问题。紧盯全市3县区、5乡镇、581村深度贫困地区,对照“一收入两不愁三保障”脱贫标准,坚持因人因户施策,制定每户脱贫计划,认真编制实施方案,摸清致贫原因、找准脱贫路子、谋划致富项目。加大倾斜支持力度,全市新增脱贫攻坚资金主要用于深度贫困地区,新增脱贫攻坚项目主要布局于深度贫困地区,新增脱贫攻坚举措主要集中于深度贫困地区,形成支持深度贫困地区脱贫攻坚的强大合力。

发展特色富民产业促增收。积极落实科技增粮措施,确保正常年景粮食总产稳定在110万吨以上。发挥产业扶贫关键作用,用足用好省上1000亿元特色产业发展专项贷款和500亿元产业发展投资基金,重点扶持农业产业化龙头企业、农民合作社和种养大户。以县区为单位加快建立产业发展公司,形成“公司+基地+专业合作社+农户”的新型产业扶贫模式,以此引领扶贫产业上规模、上档次、上水平。产业发展要在提高农产品品质、建设冷链物流、促进精深加工、打造特色品牌上下功夫,规划建设农副产品深加工园区,促进农村一二三产融合发展。新建果品基地10万亩,新增蔬菜种植面积2万亩,新建改扩建标准化规模养殖场80个,输转农村劳动力69万人,力争果品、蔬菜、畜牧产值和劳务收入分别达到108亿元、88亿元、85亿元、145亿元以上。积极推进麦积区、秦安县农村“三变”改革试点,完善贫困户与新型农业经营主体利益联结机制,让贫困群众增加更多财产性收入,分享更多加工流通增值收益。加快发展农村电子商务,积极实施电子商务进农村综合示范县项目,引导支持农民开网店、做微商,打造农产品网上交易平台。推进光伏扶贫、构树扶贫等工程,多渠道增加贫困群众收入。

改善农村基础条件补短板。用足用好省上1000亿元农村基础设施和人居环境综合治理专项贷款,加快农村基础设施、人居环境改善以及危房改造进度。新修梯田12万亩,新建堤防60公里,治理水土流失面积250平方公里,新改建农村公路3342公里,贫困地区自来水普及率达到98%。加快实施易地扶贫搬迁项目,搬迁建档立卡贫困户2214户8772人。加大教育扶贫力度,建设贫困村幼儿园110所,全面落实国家中职免学费政策,对贫困家庭中职学生发放国家助学金,为贫困家庭考入高职、本科院校的学生解决生源地信用助学贷款。推进健康扶贫,建设标准化村卫生室80个,继续全面落实“三户一孤”人员大病救助保险。把脱贫攻坚与美丽乡村建设紧密结合,加大农村污水、垃圾处理力度,绿化美化乡村环境,切实解决城乡结合部、乡镇政府所在地道路破烂和环境卫生脏乱差等问题。

完善工作推进措施强保障。坚持扶贫与扶志、扶智并重,着力激发贫困户内生动力,充分调动贫困户依靠自身能力脱贫致富的自觉性和主动性。严格落实县区党委政府脱贫攻坚主体责任和各级各部门帮扶责任,扎实落实精准扶贫各项政策措施,精准识别、精准帮扶、精准管理、精准退出,确保脱贫过程扎实、脱贫效果真实。认真执行扶贫开发投入增长机制,确保市财政当年新增财力的10%、县区财政当年新增财力的20%和各级财政当年清理收回存量资金中可统筹使用的50%以上用于精准扶贫,继续实施好“双助贷”工程,加大涉农项目资金统筹整合力度,强化扶贫资金监督管理。全面推行“双线排序比对法”,用好精准扶贫大数据管理平台,加强贫困人口动态管理,确保精准扶贫不漏一户、不漏一人。重视发挥国家定点帮扶单位和天津市东西部协作帮扶作用,在产业开发、项目资金、劳务协作、人才交流等方面争取更多支持。加大扶贫领域监督执纪问责力度,坚决防止和杜绝数字脱贫、虚假脱贫。

三、加快工业强市进程,着力促进工业经济转型升级

始终把工业作为转变方式、调整结构、培植财源、促进就业、推动发展、富民强市的支柱产业,坚定不移实施“工业强市”战略,坚持做大总量与提高质量并重、改造传统产业和培育新兴产业齐抓,加快构建现代工业体系。大力弘扬“企业家”和“工匠”精神,努力营造尊重知识、尊重人才、尊重劳动、尊重创造的良好氛围。

推动传统产业转型升级。以实施《中国制造2025甘肃行动纲要》为引领,坚持发挥优势和弥补短板双向发力,持续推进“扩量提质”工程,积极培育发展优势产业链,提升机械制造、电工电器、电子信息、医药食品、建筑材料等产业整体实力和综合效益。实施创新驱动战略,全面落实我市支持科技创新的28条具体措施,强化企业创新主体地位,推动科技成果转化运用。加快大数据、云计算、物联网与实体经济深度融合,全面提高生产技术、工艺装备、产品质量、能效环保水平,以新技术、新业态、新模式推动传统产业加快发展。实施困难企业脱困工程,帮助困难企业焕发新的生机活力。

积极培育发展新兴产业。全面实施战略性新兴产业发展规划,充分发挥骨干企业示范引领作用,重点扶持发展装配式建筑、军民融合、航空维修制造、大健康、文化旅游产品制造等“五大新兴产业”,培育工业经济发展新动能。加快天水装配式建筑产业园建设,引进实施一批装配式钢结构预制件、装配式混凝土预制件、新型建材研发生产等龙头企业和重点项目。依托市内军工企业,争取实施一批军工核心能力建设、军民用技术双向转移、军民结合产业基地等项目,推进军民融合产业快速发展。依托中梁空港城、无人机等项目,争取航空维修制造产业基地在我市布局,打造军民一体的多机种航空维修产业基地。实施发展大健康产业行动计划,以药、研、器、健、环保、能源为重点,抓好岐黄中医药产业园、张家川太极阿胶三期等项目,着力构建健康工业产业体系。推动文化创意和设计服务与关联产业融合发展,提升文化创意产业研发生产能力。

促进工业产业集聚发展。完善“一区多园”管理模式,推动开发区扩区增容,加快冀城产业园一期、东柯河工业园建设,支持县区工业园区在壮大主业、基础建设、产城融合上取得新突破。积极推动岐黄药业、铁路电缆、海辰轴仪、海林轴承等企业出城入园,促进企业向园区集中。全面落实扶持优惠政策,大力推进“小升规”“个转企”工作,力争新增规模以上企业10户以上、“个体工商户转型升级企业”500户以上。创新运用资本市场,引导支持华洋电子、长城果汁、华圆科技等条件成熟的企业尽快上市或在“新三板”挂牌。

四、坚持城乡统筹发展,着力加快区域中心城市建设

按照宜居宜游宜业的城市发展定位,统筹抓好城乡规划编制实施、城市建设管理、名城保护利用等工作,加快推进以人为核心的新型城镇化进程,力争城镇化率提高2个百分点。

重视规划编制发挥引领作用。牢固树立“规划科学是最大效益,规划失误是最大浪费,规划折腾是最大忌讳”的理念,强化规划意识,坚持规划先行,完成天水市第四版城市总体规划纲要报批,加快修编完善县城、乡镇发展规划,积极开展乡村规划编制,以科学规划引领和指导城乡建设,坚决维护规划的严肃性和权威性。

完善服务功能增强承载能力。统筹推进“八大新城”建设,优化城市布局,拓展城市空间。加快城市基础设施建设,建成成纪新城地下净水厂、固体垃圾处理循环产业园、成纪新城路网、藉河二十里铺大桥及连接线等项目,加快有轨电车示范线一期工程、天水火车站广场地下人防及商埠路、埠北路地下商业街等项目,启动实施藉河生态治理二期、新华路南延段、藉河南路西延段二期、渭河3号大桥、罗峪河景观绿化、城区重要交叉点提升改造等工程,推进三阳川城市隧道、秦州至麦积高速公路市政化改造、藉河生态治理三期、岷山片区路网、天河广场地下空间开发利用、全域无垃圾治理、羲皇大道城市风貌改造等项目,完善中心城区综合服务功能。

科学精细管理彰显城市品位。加快“智慧城市”建设步伐,促进信息资源互联共享,提升城市管理数字化、智能化水平。持续推进全国文明城市创建和园林城市建设,实施“优化城市环境、提升城市形象”工程,推进综合执法管理职能下沉和城市环卫一体化建设,着力解决交通秩序、环境卫生、占路为市、乱摆摊点等问题,采取强有力措施,下决心解决城区乱建乱搭等违法违规行为。加强社区市场和公共停车场建设,规划建设清真牛羊肉定点屠宰场,做好城区道路路面改造、背街小巷整修硬化、人行过街设施建设、公厕建设改造、城区绿化美化亮化工作,增强市政基本公共服务供给能力。实施西关片区古城保护与修复项目,加强名城名镇名村和传统村落、古树名木保护,提升历史文化名城保护利用水平。

突出特色优势发展县域经济。按照“城乡统筹、产城融合、各具特色”的思路,把产业培育作为关键支撑,依托交通区位、资源禀赋和产业基础,支持县区发展壮大工业商贸、特色农业、乡村旅游、电子商务等重点产业。把园区建设作为平台载体,加快县区现代农业产业园、科技园、创业园和田园综合体等“三园一体”建设,对县域分散的各类开发园区、工业集中区、循环经济区进行整合。把城镇建设作为重要基础,加快推进新型城镇化和特色小镇建设,抓好麦积区甘泉民俗风情小镇、百花小镇、峡门风情小镇,秦州区娘娘坝丝路风情小镇、罗玉生态小镇、孙集文化小镇,甘谷礼辛风情小镇,秦安刘坪生态小镇,武山滩歌民俗文化小镇,清水上邽水镇、张家川马鹿特色旅游小镇建设。把改革创新作为动力源泉,深化县级财税体制、金融市场、土地管理等领域改革,推进科技创新、市场创新、管理创新、制度创新,激发县域经济发展内生动力。

五、协调发展社会事业,着力提高公共服务均等化水平

坚持普惠性、保基本、均等化、可持续方向,协调推进教育、文化、体育、卫生等社会事业发展,努力让全市人民享有更优质更公平的基本公共服务。

积极推动教育均衡发展。继续实施城区教育资源提升“2521”计划,全力抓好全面改薄、贫困村幼儿园和市三中、玉泉中学、人民路小学迁建等项目,改善教育基础条件。实施第三期学前教育行动计划,扩大城乡学前教育资源,加强幼儿园规范管理,提升学前教育发展水平。加快义务教育标准化学校建设,优化城乡教育资源布局,逐步解决“大班额”和“择校热”问题,确保秦安县、清水县提前完成县域义务教育均衡发展达标任务。加大普通高中特色实验校和示范校建设力度,提高高中阶段教育质量和普及水平。加强教师队伍建设,落实乡村教师支持计划,逐步提高农村教师和艰苦地区教师待遇水平。优化职业教育结构布局,启动建设天水职业教育园区,筹建天水职业技术学院及附属中小学、幼儿园。支持高等教育发展,推动天水师范学院升格天水师范大学。

大力发展文化体育事业。深入实施文化惠民工程,新建综合文化服务中心32个、街道综合文化站3个,继续做好“三馆”免费开放,充分发挥“乡村舞台”“文化集市”作用,丰富群众性文化活动。积极推进“历史再现”工程,加强文化遗产保护利用,改造提升张家川、清水等4个博物馆,加快大地湾考古遗址公园、黄河雕漆文化博览园等项目建设。加强文艺精品创作,推出文艺天水系列丛书,推动《一画开天》《麦积圣歌》《大秦文公》等精品剧目在旅游旺季实现常态化演出,谋划创作一批新的舞台、影视剧目。大力开展全民健身活动,完成市体育中心建设,新建乡镇及社区体育健身中心8个、行政村体育惠民工程80个,做好省第十四届运动会训练参赛工作。

提升医疗卫生服务水平。加快健康天水建设,深化公立医院综合改革,实施改善医疗服务行动计划,建成市县全民健康信息平台,促进医疗服务规范化、标准化。健全完善分级诊疗服务体系,加强医联体及重点专科建设,推进医师多点执业和家庭医生签约服务,提高基层医疗服务能力。全面落实药品采购“两票制”,让群众用到价格低廉、安全放心的药品。坚持中西医并重,实施中医药基层能力提升工程,加快建设多层次养老服务体系,推动医疗卫生和养老服务融合发展。进一步加强计生服务、妇幼保健工作,广泛开展爱国卫生运动,提高全民医疗保障水平。

六、重视保障改善民生,着力增强群众幸福感和获得感

坚持以人民为中心的发展思想,以满足人民群众日益增长的美好生活需要为目标,努力在劳有所得、老有所养、住有所居、弱有所扶等方面取得新进展。

努力提高就业质量。把就业作为最大的民生,落实就业创业扶持政策,实施“高校毕业生就业促进计划”和“大学生创业引领计划”,提升创业孵化基地、创业园区和就业见习基地建设质量和层次,促进大众创业万众创新。加强就业创业技能培训和服务引导,积极为创业人员提供担保贴息贷款,做好公益性岗位托底安置工作,促进高校毕业生、转业军人、农民工、零就业家庭等群体多渠道就业创业,城镇登记失业率控制在4%以内。加强劳务基地建设,培养扶持职业中介组织、劳务带头人、劳务经纪人,提升劳务输转组织化程度。高度重视人才工作,统筹做好人才培养、引进、使用,最大限度激发各类人才的创新创造活力。

完善社会保障体系。按照兜底线、织密网、建机制的要求,提高城市低保和农村低保一、二类对象补助水平及农村特困人员救助供养补助标准,完成全民参保登记工作和机关事业单位养老保险制度改革,做好城乡居民和被征地农民养老保险工作。加强“五险合一”社会保险信息化建设,完善城乡居民基本医疗保险运行机制,实现基本养老、基本医疗、失业、工伤、生育等保险法定人员全覆盖。加大社会保险费欠费征缴力度,确保社会保险基金安全可持续。大力发展社会福利和慈善事业,做好农村留守妇女、儿童和空巢老人、特困群体关爱服务工作。坚持房子是用来住的、不是用来炒的定位,加快建立多主体供给、多渠道保障、租购并举的住房制度,实施棚户区改造项目57个2.58万户,加大公共租赁房建设力度,逐步解决城镇低收入群体住房困难。加快麦积全国综合养老示范基地建设,规范城市日间照料中心等养老机构管理,提升养老服务水平。重视殡葬改革和丧葬场地建设,启动市殡仪馆、市殡葬管理所搬迁新建,开工建设麦积区曹温梁公墓区。

创新加强社会治理。深入开展“七五”普法,增强市民法治意识,加快依法治市进程。启动实施“雪亮工程”,完善立体化社会治安防控体系,依法严厉打击黄赌毒黑拐骗等违法犯罪活动。严格落实“党政同责、一岗双责、齐抓共管、失职追责”安全生产责任制,细化强化企业安全生产主体责任,坚决遏制重特大安全事故。实施食品安全战略,落实“四个最严”要求,保障群众饮食用药安全。打好防范化解重大风险攻坚战,重点加强金融风险防控,坚决打击非法集资、电信诈骗活动。完善预防和化解社会矛盾机制,做好人民调解和法律援助工作,落实信访工作责任制,办好“市委书记市长留言板”“市长信箱”。推进双拥共建工作,争创全国双拥模范城“六连冠”。重视做好民族宗教工作,开展民族团结进步创建活动,加快民族地区发展。加强统计调查、档案史志、防震减灾、人民防空、社会团体、妇女儿童、社区管理、老龄老干、老年教育、残疾人事业和关心下一代工作。

七、加强生态环境保护,着力推动绿色低碳循环发展

牢固树立绿水青山就是金山银山的理念,实行最严格的生态环境保护制度,推动形成绿色发展方式和生活方式,努力使天水的天蓝、山绿、水清、生态环境美好。

全面加强生态建设。积极实施生态系统保护和修复工程,加强自然保护区、水资源保护区、风景名胜区生态治理,提升生态系统质量和稳定性。高度重视造林绿化工作,把重点放在现有果园的补植补栽更新换代、城区乡镇村组人居环境的绿化、交通沿线旅游景区绿化美化等方面。认真实施新一轮退耕还林、天然林保护、“三北”五期防护林暨泾渭河流域百万亩防护林基地建设、中央森林生态效益补偿、森林抚育等生态工程,抓好南北两山50公里绿色生态长廊建设和铁路公路沿线绿化美化,完成营造林20万亩。开展生态保护红线、永久基本农田、城镇开发边界三条控制线划定工作。推进生态环境监管体制改革,坚决制止和惩处破坏生态环境行为。

从严治理环境污染。严格落实环境保护“八项机制”和污染治理“十大工程”,持续推进大气、水、土壤污染防治“三大战役”,巩固扩大中央环保督察反馈问题整改成效。加强大气污染防治,抓好散煤燃烧治理、燃煤锅炉淘汰改造、城市扬尘管控、低空面源污染治理、城区环境网格化监管等工作。推进水污染防治,落实“河长制”责任体系,强化城市生活污水收集治理,加大渭河、藉河、葫芦河、散渡河等重点河流整治力度,全面消除黑臭水体。加快土壤污染防治,扩大轮作休耕试点,完成“禁养区”养殖企业和专业养殖户搬迁取缔,建立完善地膜回收长效机制。常态化开展全域无垃圾治理,实施农村环境连片整治项目,加大环卫设施建设力度,逐步建立“农户分类、村组收集、乡镇转运(处理)、集中处理”的农村垃圾治理体系。

推动绿色低碳发展。加快发展绿色金融,积极推广利用地热、地岩热取暖技术等,大力发展节能环保、清洁生产和清洁能源产业,推动建立绿色低碳循环发展的经济体系。办好绿色及可持续发展麦积山论坛,培育新能源、新材料等产业企业。深入实施《天水市循环经济发展规划》,加快天水装备制造循环经济基地建设,推广天水国家农业科技园区循环发展模式,促进资源节约循环利用。持续降低工业、建筑业等领域能耗物耗,开展节水行动,全面完成节能降耗目标任务。倡导简约适度、绿色低碳的生活方式,积极开展节约型机关和绿色家庭、绿色学校、绿色社区创建活动。

八、持续深化改革开放,着力激发经济社会发展活力

深入推进各项改革,全方位扩大对外开放,不断激发全社会创造动力和发展活力,努力实现更高质量、更有效率、更加公平、更可持续的发展。

深化重点领域改革。进一步理清市区政府城市规划建设管理运营的职责,切实减少推诿、扯皮、掣肘和越位、错位、缺位等问题,促进城市建设管理工作高效协调运行。完善国有资产管理运行机制,整合组建城投、工投、交投、农投、水投、机投、文旅投七大投融资公司,优势互补、资源共享,做大做强投融资平台,并纳入市政府国资委统一监督管理;整合市级政策性融资担保公司,壮大担保力量。持续推进“接放管服”改革,精简优化审批事项,大力推行“互联网+政务服务”,实现投资项目在线审批监管平台与政务服务网深度联接、全程监管,市县区“双随机、一公开”“双告知、一承诺”监管全覆盖。全面推行企业登记全程电子化和电子营业执照,实施市场准入负面清单制度,支持民营企业发展,放宽服务业准入限制,新增市场主体8000户以上。探索承包地“三权”分置有效实现形式,深化农村集体产权制度改革,加快推进农村“三变”改革,发展多种形式农业适度规模经营。深化国资国企改革,稳步推进国有企业“三供一业”分离移交工作,大力发展混合所有制经济。严格政府性债务管理,积极探索财政资金撬动银行贷款和社会资本的模式和途径,充分利用政府债券、PPP模式、基金模式等筹集项目建设资金。发展股权投资基金、金融租赁公司、金融消费公司等新型金融机构,提升金融服务保障能力。

加强对外交流合作。充分利用宝兰高铁带动开放开发的有利条件,全面加强与“一带一路”、中新南向通道、关中—天水经济区、陇海兰新经济带沿线城市和地区的经贸合作、人文交流,力争在更大范围、更广领域和更高层次融入区域发展大局。精心筹办好公祭伏羲大典、影像丝绸之路·天水国际摄影双年展、中国龙舟公开赛、麦积山全国山地自行车邀请赛、国际传统射箭锦标赛等文化体育活动,让更多的人了解天水、走进天水、投资天水。

努力扩大对外贸易。充分发挥兰州海关天水办事处便利企业通关、降低进出口成本等有利条件,积极推进天水铁路口岸、保税物流中心建设,大力发展跨境电商,支持华天电子、华洋电子、海林轴承、美通公司、易天果业、成纪药业等企业开拓国际市场,努力扩大电子电工、装备制造、特色农产品、清真食品出口规模,提升外向型经济发展水平,完成外贸进出口总额32.5亿元。

持续推进品牌建设。扎实开展“全国质量强市示范城市”创建活动,抓好全国电工电器产业知名品牌创建示范区和75个“质量强市”示范点建设,筹建“天水商标品牌展示馆”,扶持引导企业培育注册文化旅游、现代服务业和涉农商标。深入开展马德里体系国际商标注册,加强地理标志产品保护、农产品地理标志证明商标注册和农产品地理标志保护产品认证,新增注册商标500件以上,争创中国驰名商标2个、甘肃省名牌产品9个。

各位代表,群众利益无小事,人民群众对美好生活的向往就是政府工作的奋斗目标。2018年,市政府将继续为群众办好10件民生实事:(1)建成秦州热源厂二期、亚行贷款城区供热管网改造二期和麦积桥南集中供热扩容改造项目,新增城区集中供热面积600万平方米以上。(2)加快智慧城市建设步伐,实现城区广场、公园、机场、车站、宾馆、饭店、商场、医院、旅游景点等公共场所无线网络全覆盖和免费上网。(3)积极推进“厕所革命”,新改建城区厕所52座,旅游景区厕所48座,交通沿线厕所23座。(4)实施城中村、城郊村和社区改灶、改炕、改炉、优质煤炭补贴“三改一补”3万户,打好蓝天保卫战。(5)实施棚户区改造25000户,改造农村C级危房13000户。(6)优化市区交通管理与设施,新建城区人行过街天桥4座,新增城区停车位3000个,整修城区主次干道5万平方米,着力解决交通拥堵、停车难等问题。(7)引导1000名高校毕业生进企业服务,进一步提升就业服务质量和水平。(8)高度重视被征地农民养老保险政策落实,切实解决失地农民养老问题。(9)完成市三中迁建,确保秋季实现招生,进一步缓解城区“入学难”问题。(10)继续为全市69.3万名“三户一孤”人员办理大病救助保险。

全面加强政府自身建设

新时代新任务,对政府自身建设提出了新的更高要求。我们将坚持不懈加强政府自身建设,依法行政、从严治政、高效施政、廉洁从政,努力建设人民满意的政府。

坚持理论武装,建设学习型政府。完善市政府党组中心组学习制度,推进“两学一做”学习教育常态化制度化,认真开展“不忘初心、牢记使命”主题教育,深刻学习领会党的十九大精神和习近平新时代中国特色社会主义思想,深入贯彻习近平总书记视察甘肃重要讲话和“八个着力”重要指示精神,切实增强政治意识、大局意识、核心意识、看齐意识。加强调查研究,深化市情认识,创造性贯彻落实省委、省政府和市委的各项决策部署。重视加强政府工作人员教育培训,学习新政策、掌握新知识、增强新本领,切实解决“知识恐慌”“本领恐慌”等问题,不断提升做好各项工作的能力。

坚持依法行政,建设法治型政府。重视地方立法工作,及时向市人大常委会提出地方立法项目建议,认真做好以市政府作为提案人的地方性法规(草案)的论证起草和提请审议工作,根据需要做好政府规章的制定出台工作。全面贯彻《法治政府建设实施纲要》,落实市政府常务会议会前学法制度,严格按法定权限和程序履行职责,坚持重大行政决策公众参与、专家论证、风险评估、合法性审查、集体讨论决定及重大行政决策实施后评估等制度,推动决策科学化、民主化、法治化。落实政府新闻发布制度,全面推进政务公开,推动政府法律顾问制度全覆盖。自觉接受市人大及其常委会的法律监督和工作监督、市政协的民主监督,认真听取民主党派、工商联和社会各界意见建议,提高人大代表建议和政协提案办理质量。

坚持履职为民,建设服务型政府。政府的一切工作都要充分体现以人民为中心的发展思想,始终把人民的期盼作为政府工作的目标,关心群众疾苦、倾听群众呼声、了解群众意愿,力求政府做出的工作部署、出台的政策措施更加符合人民群众意愿。牢固树立“人民政府为人民”的理念,在任何时候、任何情况下都把群众利益放在第一位,做到财力向民生集中、工作向民生倾斜、项目向民生聚焦、服务向民生覆盖,千方百计保基本、补短板、兜底线、促公平,尽力把顺民意、解民忧、暖民心的实事办好、好事办实,让人民群众共享改革发展成果。

坚持务实高效,建设效能型政府。始终把加快发展作为第一要务,把干好事业作为第一追求,对照重点工作部署和主要指标任务,一项一项分解细化,一项一项盯紧落实,强化督促检查,严肃问责追责,确保中央和省市决策部署落地见效。开展政务诚信建设年活动,建立守信激励与失信惩戒机制,加大政务失信行为惩戒力度,不断提高政府执行力和公信力。加强电子政务信息平台建设,积极推行无纸化办公,提升行政工作效能。严格执行中央“八项规定”精神和省市委关于作风建设的规定和要求,积极践行“三严三实”,持之以恒纠正“四风”,坚决整治庸懒散软拖等不作为、慢作为、乱作为现象,推动政风行风持续好转。

坚持勤政廉政,建设廉洁型政府。市政府一班人要发挥“头雁”效应,以身作则、以上率下,严格落实中央和省市委廉洁从政各项规定。全面落实党风廉政建设主体责任和领导干部“一岗双责”,健全廉政风险防控制度,加强对重点领域、关键环节、重要岗位权力运行的监督制约,进一步规范政府采购、土地出让、矿产开发、工程招投标、企业改革、财政资金管理、扶贫资金使用等工作,加强行政监察、财政监管和审计监督,实行“一案双查”,严肃查处违纪违法案件。厉行勤俭节约,“三公”经费只减不增。健全农村“三资”管理制度,扎实开展“三纠三促”“两查两保”等专项行动,坚持无禁区、全覆盖、零容忍,严肃查处损害群众利益的人和事,树立为民务实清廉的良好形象。

各位代表,新起点孕育新希望,新时代要有新作为。我们要紧密团结在以习近平同志为核心的党中央周围,在省委、省政府和市委的坚强领导下,认真学习贯彻党的十九大精神,不断强化“四个意识”,不忘初心、牢记使命,扎扎实实做好政府各项工作,为坚决打赢脱贫攻坚战、决胜全面建成小康社会、建设幸福美好新天水而努力奋斗!
